# Temperate phage-antibiotic synergy across antibiotic classes reveals new mechanism for preventing lysogeny

**DOI:** 10.1128/mbio.00504-24

**Published:** 2024-05-17

**Authors:** Amany M. Al-Anany, Rabia Fatima, Gayatri Nair, Jordan T. Mayol, Alexander P. Hynes

**Affiliations:** 1 Department of Biochemistry and Biomedical Sciences, McMaster University, Hamilton, Ontario, Canada; 2 Department of Medicine, McMaster University, Hamilton, Ontario, Canada; 3 Farncombe Family Digestive Health Research Institute, McMaster University, Hamilton, Ontario, Canada; 4 Michael G. DeGroote Institute for Infectious Disease Research, McMaster University, Hamilton, Ontario, Canada; University of Wisconsin-Madison, Madison, Wisconsin, USA; Baylor College of Medicine, Houston, Texas, USA

**Keywords:** bacteriophage, temperate phage, lysis-lysogeny, phage-antibiotic synergy, antimicrobial resistance

## Abstract

A recent demonstration of synergy between a temperate phage and the antibiotic ciprofloxacin suggested a scalable approach to exploiting temperate phages in therapy, termed temperate phage-antibiotic synergy, which specifically interacted with the lysis-lysogeny decision. To determine whether this would hold true across antibiotics, we challenged *Escherichia coli* with the phage HK97 and a set of 13 antibiotics spanning seven classes. As expected, given the conserved induction pathway, we observed synergy with classes of drugs known to induce an SOS response: a sulfa drug, other quinolones, and mitomycin C. While some β-lactams exhibited synergy, this appeared to be traditional phage-antibiotic synergy, with no effect on the lysis-lysogeny decision. Curiously, we observed a potent synergy with antibiotics not known to induce the SOS response: protein synthesis inhibitors gentamicin, kanamycin, tetracycline, and azithromycin. The synergy results in an eightfold reduction in the effective minimum inhibitory concentration of gentamicin, complete eradication of the bacteria, and, when administered at sub-optimal doses, drastically decreases the frequency of lysogens emerging from the combined challenge. However, lysogens exhibit no increased sensitivity to the antibiotic; synergy was maintained in the absence of RecA; and the antibiotic reduced the initial frequency of lysogeny rather than selecting against formed lysogens. Our results confirm that SOS-inducing antibiotics broadly result in temperate-phage-specific synergy, but that other antibiotics can interact with temperate phages specifically and result in synergy. This is the first report of a means of chemically blocking entry into lysogeny, providing a new means for manipulating the key lysis-lysogeny decision.

**IMPORTANCE:**

The lysis-lysogeny decision is made by most bacterial viruses (bacteriophages, phages), determining whether to kill their host or go dormant within it. With over half of the bacteria containing phages waiting to wake, this is one of the most important behaviors in all of biology. These phages are also considered unusable for therapy because of this behavior. In this paper, we show that many antibiotics bias this behavior to “wake” the dormant phages, forcing them to kill their host, but some also prevent dormancy in the first place. These will be important tools to study this critical decision point and may enable the therapeutic use of these phages.

## INTRODUCTION

The ongoing crisis of antimicrobial resistance has rekindled interest in bacteriophage (phage) therapy as an alternative to antibiotics, as these bacterial viruses may soon be one of the few remaining options to clear bacterial infections (1). Phages are often administered alongside antibiotics. This is largely because phages must prove themselves alongside the standard of care—antibiotics—but also guided by the idea that multiple selective pressures will decrease the emergence of resistance (2). This combination has led to the discovery that the two components, phage and antibiotics, can interact to increase their efficacy, termed phage-antibiotic synergy (PAS).

The term PAS was first coined in 2007 when sub-inhibitory concentrations of the β-lactam cefotaxime (3) resulted in enlarged plaques of the virulent *Escherichia coli* phage phiMFP, an increase in phage production, and an increase in the latency period (3, 4). In contrast, using aztreonam lysine in combination with virulent phages E79 and phiKZ, Davis et al. (5) demonstrated PAS characterized by a decrease in infection latency, burst size, and accelerated lysis. Several studies have suggested that the bacterial SOS response plays a minor role and that PAS is a result of an alteration in cell morphology in response to the action of these antibiotics (3, 4). PAS appears to be broadly applicable, spanning phages across the myovirus (3, 6–15), siphovirus (16, 17), and podovirus morphologies (7, 18–22). PAS has also been demonstrated in many hosts: *Klebsiella pneumoniae* (22–24)*, Pseudomonas aeruginosa* (8, 25–30)*, Acinetobacter baumannii* (7)*, Staphylococcus aureus* (10, 31–36), and *E. coli* (37–39)*,* as well as across antibiotics of multiple classes including β-lactams (3, 6, 23, 28, 29, 33, 39), fluoroquinolones (3, 9, 16, 18, 25, 26, 29), aminoglycosides (16, 26, 27, 30), and tetracyclines (33).

In all of these studies, PAS was in the context of virulent (strictly lytic) phages, as the lysogenic life cycle of temperate phages is considered an insurmountable hurdle for therapy. While temperate phages have proven necessary—and successful—in therapy, they have been genetically modified to prevent lysogeny (40). This is primarily because, during lysogeny, the phage integrates its genome into the host and as a result will not have an immediate bactericidal effect (41). Furthermore, this cycle affects host fitness and may leave the host with more virulent traits via the integrated prophage, in addition to causing horizontal gene transfer by transduction (40). However, transduction is also common in virulent phages (41) and depends more on the packaging mechanism of the phage than its life cycles (42). Most importantly, lysogeny will typically result in protection from superinfection (43). While any antibiotic will select for resistance, a temperate phage will also generate it. This makes lysogeny prevention key to the eventual therapeutic success of temperate phages.

Temperate phages usually remain quiescent in the cell unless exposed to a stressor that results in irreversible switching to a lytic cycle (44). This awakening of dormant phages is known as induction (45) and can happen either spontaneously (46) or as a result of external stressors (44). Our understanding of prophage induction primarily stems from well-characterized models of lambda and lambdoid (lambda-like) lysogens (47, 48). We have recently shown that co-administration of a temperate phage HK97 with the fluoroquinolone antibiotic ciprofloxacin below minimum inhibitory concentrations (MIC) yields potent synergy resulting in bacterial eradication (≥9.7 × 10^7^-fold reduction) (49). This synergy does not greatly increase final phage titers, latency period, or burst size; instead, it greatly reduces the rate of lysogeny. As such, it is distinct from traditional PAS. We coined the term temperate phage-antibiotic synergy (tPAS) for synergy with antibiotics that specifically exploits the lysis-lysogeny decision (48).

As the interaction between temperate phages and stressors such as ciprofloxacin is known to be widespread across SOS-inducing antibiotics (3, 50–53), we hypothesized that tPAS could result from the activities of other antibiotics. Demonstrating this may enable a safe approach to allow for the use of these phages in therapy, potentially more scalable than genetic modification used previously.

## RESULTS AND DISCUSSION

### Generalizability of tPAS across different quinolones

The standard to test synergy across antibiotics is a checkerboard assay (54), also referred to as synograph (38). This technique previously revealed that tPAS reduced the effective ciprofloxacin MIC despite the poor effectiveness of the phage-alone condition (48).

**Fig 1:**
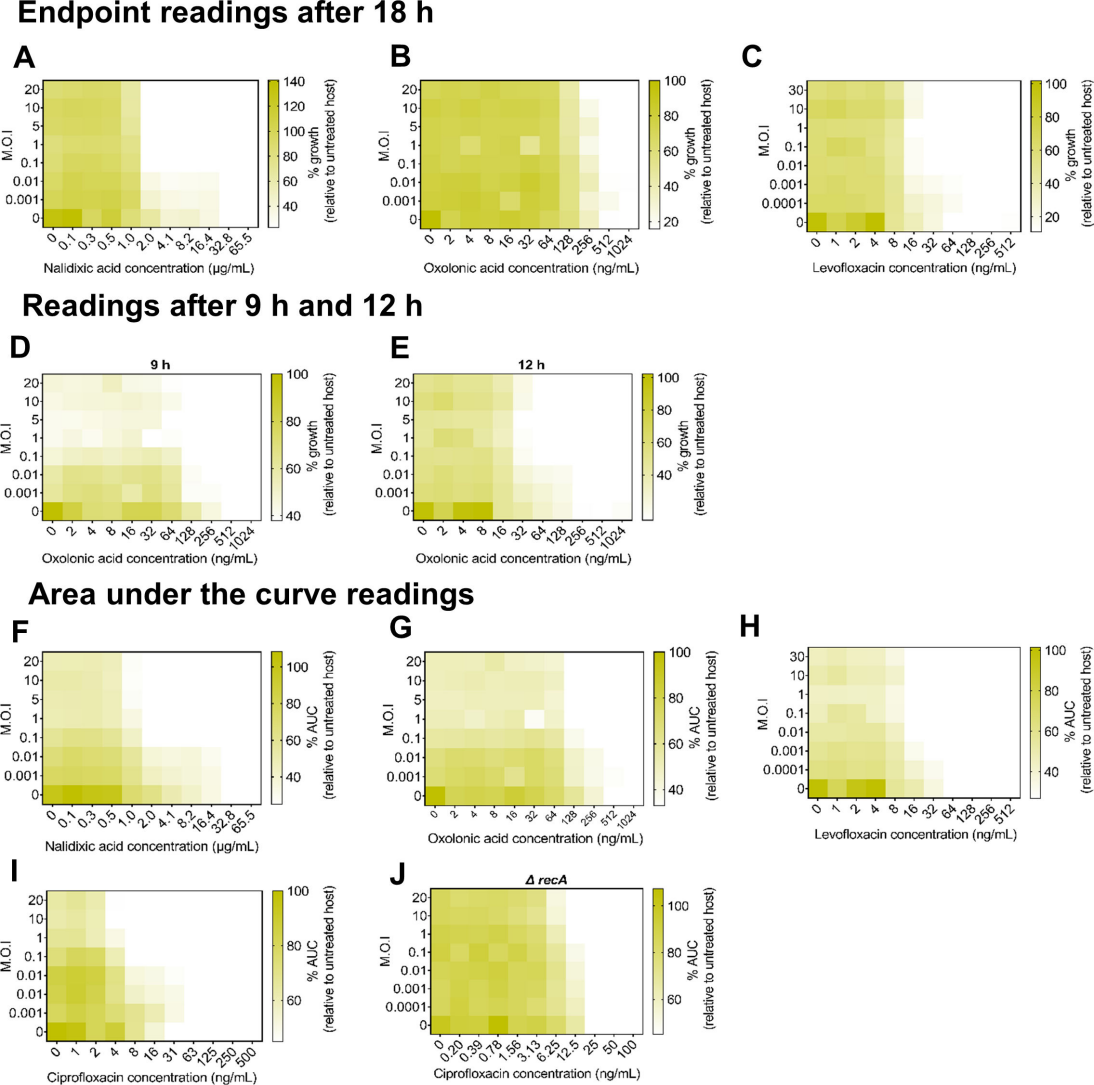
Temperate phage-antibiotic synergy across quinolones. Checkerboard assay of HK97 with quinolones: (**A**) nalidixic acid, (**B**) oxolinic acid, and (**C**) levofloxacin. Endpoint growth relative to untreated bacterial control, averaged among three biological replicates, plotted as a heatmap. Readings at 9 and 12 h. Checkerboard assay of HK97 and oxolinic acid after 9 h (**D**) or 12 h (**E**). Growth relative to untreated bacterial control, averaged among three biological replicates, plotted as a heatmap. Area under the curve readings. Checkerboard assay of HK97 and (**F**) nalidixic acid, (**G**) oxolinic acid, (**H**) levofloxacin, and (**I**) ciprofloxacin. Area under the curve relative to untreated bacterial control, averaged among three biological replicates, plotted as a heatmap. (**J**) Checkerboard assay of HK97 and ciprofloxacin in *recA* mutant. Area under the curve relative to untreated bacterial control, averaged among three biological replicates, plotted as a heatmap.

To test whether the previously reported synergy was generalizable across other antibiotics, we challenged the same *E. coli* K-12-HK97 phage-host pairing used to establish the existence of tPAS (48). We first sought to establish whether the reported synergy held true across other antibiotics in the same class as ciprofloxacin and therefore performed checkerboard assays with three other quinolones: nalidixic acid, oxolinic acid, and levofloxacin. In these assays, the cutoff value for MIC was calculated for each antibiotic based on antibiotic-alone growth curves (Fig. S1), with any percent growth value less than or equal to that obtained with the MIC colored white. Nalidixic acid and phage HK97 yielded a 16-fold reduction in MIC at almost all tested multiplicity of infection (MOIs) (Fig. 1A). Curiously, there was a far weaker synergy with oxolinic acid and levofloxacin (Fig. 1B and C), resulting in only two- and fourfold reductions in MIC, respectively.

The observation that different antibiotics within the same class result in drastically different synergy profiles concerned us. The SOS-inducing effect of these antibiotics is well documented (55, 56), as is the association between SOS induction and phage induction (57, 58). We hypothesized that the synergy might be occurring but obscured by the endpoint due to the emergence of resistant mutants. Examining growth with oxolinic acid at two earlier time points, 9 h (Fig. 1D) and 12 h (Fig. 1E), revealed a clear synergistic effect resulting in a potent 16-fold reduction in MIC whose effects are obscured over time by bacterial regrowth. We opted to monitor continuously (Fig. S2) and represent our data as the area under the curve (AUC) to capture cumulative dynamics of phage antibiotic interaction over time. In these assays, the percent AUC corresponding to the well determined MIC (Fig. S1) was set as a threshold for white color. When the heat maps were plotted as a function of percent AUC, we observed a synergistic effect resulting in a 32-fold reduction for nalidixic acid (Fig. 1F), a fourfold reduction in MIC for both oxolinic acid and levofloxacin (Fig. 1G and H), and, repeating our prior assays for ciprofloxacin in this manner, an eightfold reduction for ciprofloxacin (Fig. 1I) that was lost in a *recA* background (Fig. 1J).

Overall, the use of quinolones resulted in synergy but with different temporal patterns based on the ability of the antibiotic to prevent the long-term regrowth of bacterial survivors.

### Generalizability of tPAS across different antibiotic classes

**Fig 2:**
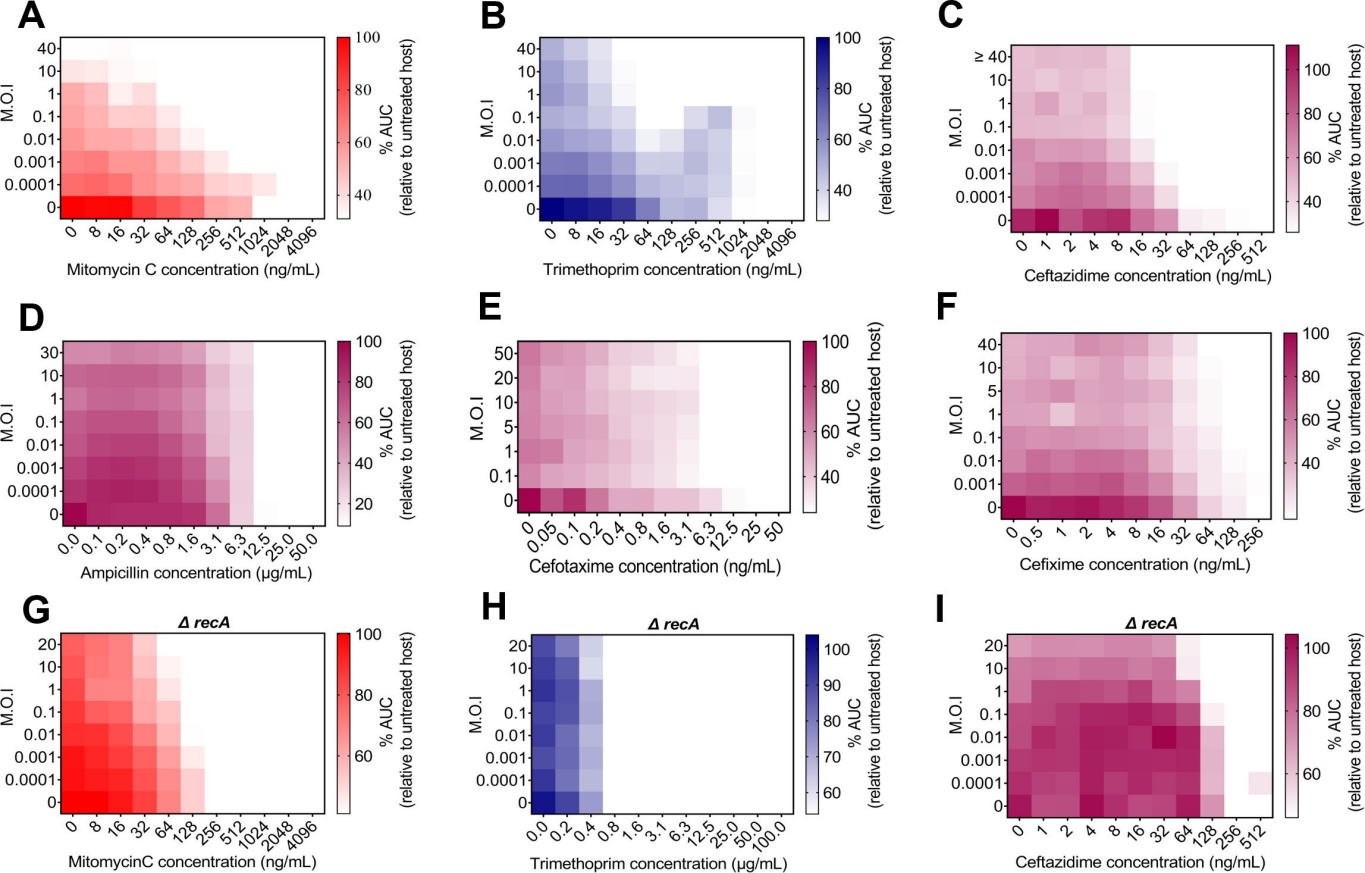
Temperate phage-antibiotic synergy across SOS-inducing antibiotics. Checkerboard assay of HK97 (**A**) mitomycin C, (**B**) trimethoprim, (**C**) ceftazidime, (**D**) ampicillin, (**E**) cefotaxime, and (**F**) cefixime. Color is kept consistent across drugs of the same class. Area under the curve relative to untreated bacterial control, averaged among three biological replicates, plotted as a heatmap. Checkerboard assay of HK97 and (**G**) mitomycin C, (**H**) trimethoprim, and (**I**) ceftazidime in *recA* mutant. Area under the curve relative to untreated bacterial control, averaged among three biological replicates, plotted as a heatmap.

Armed with a methodology more sensitive to temporal variations, we moved to investigate tPAS across other phage-inducing agents, conducting checkerboards with two other antibiotic classes that result in DNA damage: mitomycin c and trimethoprim. Mitomycin inhibits DNA synthesis and is well known as an inducer of temperate phages like lambda and HK97 (51, 52). Trimethoprim is a sulfa drug that inhibits thymine synthesis by targeting dihydrofolate reductase, which inhibits folic acid synthesis, leading to DNA damage (52, 59). It has been shown to induce temperate phages in *Staphylococcus aureus* (60). In our mitomycin C checkerboards (Fig. 2A), we observed synergy like that seen for ciprofloxacin, as expected. This effect resulted in a peak of a 64-fold decrease in mitomycin C MIC at the highest ineffective concentration of phage utilized. While we observed a clear synergistic effect with trimethoprim (Fig. 2B), resulting in an effective 32-fold decrease in trimethoprim MIC, unlike for other antibiotics, synergy was only apparent at phage MOIs higher than 1. For these known SOS-inducing antibiotics, synergy was drastically reduced in the *recA* mutant (Fig. 2G and H).

β-Lactams are another well-characterized class of antibiotics. They inhibit peptidoglycan synthesis by binding to a set of membrane proteins known as “penicillin-binding proteins” (61). The presence of β-lactams is reported to induce the SOS response (62, 63) and consequently induce phages through the DpiBA two-component signal transduction system (62). We saw no clear synergy with ampicillin (Fig. 2D), and at best, a weak twofold reduction in MIC with cefotaxime (Fig. 2E), improving to a fourfold reduction for cefixime at higher MOIs (Fig. 2F). In contrast, we observed a clear synergistic effect with ceftazidime (Fig. 2C), resulting in a 16-fold reduction in MIC.

Given that β-lactams displayed inconsistent synergy, we hypothesized that it might be dependent on the extent to which each antibiotic induces the SOS response. First, to confirm the SOS response was involved, we tested the antibiotic showing the clearest synergy (ceftazidime) in our *recA* mutant. Because RecA plays a role in antibiotic resistance, we first determined the MIC of each drug in our *recA* mutant (Fig. S3). As with ciprofloxacin (Fig. 1J), in the *recA* background, almost all synergy was lost for ceftazidime (Fig. 2I). Wherever we see strong synergy, that synergy is consistently *recA* dependent.

### Assessment of *recA* activation by antibiotics displaying synergy

To examine whether the extent of SOS activation plays an important role in our observed synergy, we studied *recA* and *sulA* gene expression across antibiotic challenges. The SOS response initiates with the activation of the regulatory protein RecA, which will polymerize on ssDNA and consequently induce the autocleavage of LexA (50). RecA regulates its own gene expression (64), and LexA cleavage also increases *recA* transcription (65), so *recA* expression is a good proxy for early SOS activation. In contrast, *sulA* is only induced in the later stages and/or in the presence of substantial DNA damage (66).

**Fig 3:**
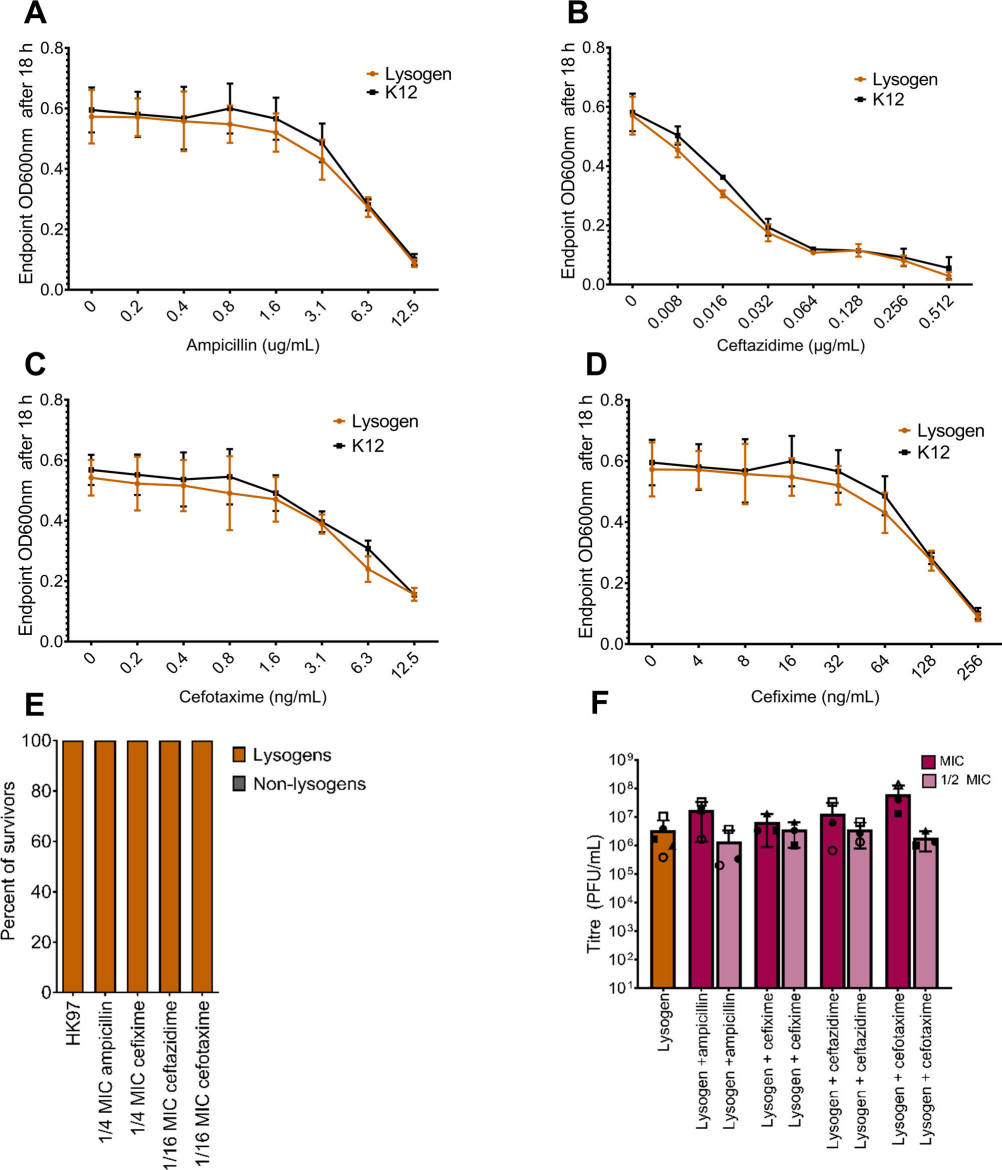
HK97 lysogen sensitivity to β-lactams and lysogeny frequency. MIC in liquid culture for wild-type *E. coli* K-12, and lysogen control was tracked after challenging with serial dilutions of (**A**) ampicillin, (**B**) ceftazidime, (**C**) cefotaxime, and (**D**) cefixime (means ± SD, *n* = 3 biological replicates, each with three technical replicates). (**E**) Percentage of lysogen and non-lysogen survivors from 20 colonies after overnight HK97 and β-lactams challenges averaged. Concentrations were selected where we have seen the highest difference in AUC between phage challenge and phage + antibiotic treatment. (**F**) Phage quantification after 18 h when unchallenged and when challenged with MIC and 1/2 MIC concentrations of ampicillin, cefixime, ceftazidime, and cefotaxime, (means ± SD, *n* = 3 biological replicates). Significance for panel F was calculated using two-way ANOVA with no significant difference. Shapes represent different biological replicates.

We employed an engineered promoter-reporter gene construct that expresses green fluorescent protein (GFP) upon *recA* or *sulA* expression (67, 68), normalizing the fluorescence to bacterial growth. As expected, we observed a significant fold increase in *recA* expression with known SOS-inducing antibiotics (Fig. S4A) and no change with gentamicin (Fig. S4C), which is not known to induce the SOS response. Interestingly, in β-lactam-treated cultures, we observed the least fold change in both *recA and sulA* expression (Fig. S4A and B), and this held true regardless of the extent of synergy caused by the β-lactam (Fig. 2C through F),

Overall, we could not attribute the stronger synergistic effect in ceftazidime to a higher stimulation of the SOS response. To confirm that the synergistic effect seen in some β-lactams is mechanistically distinct from the synergy seen with other SOS-inducing agents, we investigated whether the observed synergy influenced the lysis-lysogeny decision. Our examination included assessing the sensitivity of an HK97 lysogen to β-lactams compared to wild-type *E. coli* K-12. Contrary to the effect seen with ciprofloxacin (49), the lysogen exhibited no differential sensitivity, indicative of induction, to any of the four β-lactams (Fig. 3A through D). Critically, the survivors of PAS challenges showed no change in the frequency of lysogens compared to culture challenged with phage alone (Fig. 3E), indicating that the observed synergy in some β-lactams is mechanistically different from tPAS and does not appear to select against the formation of lysogens. Furthermore, the HK97 titer did not increase significantly over spontaneous induction after 18 h for all four β-lactams across a variety of concentrations (Fig. 3F), indicating a lack of induction.

We hypothesize that the synergy with certain β-lactams is traditional PAS, independent of the lysis-lysogeny decision, and potentially driven by cell filamentation (3, 69). Prior work with phage T4 in a *recA* mutant established that the SOS response is involved in traditional PAS (3). Moreover, our synergistic effect in β-lactams matches the result of Uchiyama et al. (70), where ceftazidime showed the highest PAS compared to other tested β-lactams when combined with different virulent phages infecting *P. aeruginosa*. Wiegand et al. (71) demonstrated that both ceftazidime and cefixime antibiotics induce filamentation in *E. coli*. Additionally, the study noted that ceftazidime produces large filaments in *Plesiomonas shigelloides*, although it does not specify their size in comparison to cefixime. The variability in the efficacy of different β-lactams could be attributed to their varying affinities for different PBPs, leading to distinct enzymatic reactions and morphological changes (72–74).

### tPAS with non-SOS-inducing antibiotics

Since ceftazidime had low SOS activation yet demonstrated reasonable synergy—a profile matching PAS rather than tPAS—we wanted to ensure gentamicin, which did not detectably induce SOS response in our assays (Fig. S4A and B), would not result in synergy. Aminoglycosides like gentamicin are not known to induce phages (75), and, might even antagonize the phage by inhibiting protein synthesis upon which they depend (76). In fact, a recent paper demonstrated that kanamycin impaired phage infection by temperate phage lambda (77).

**Fig 4:**
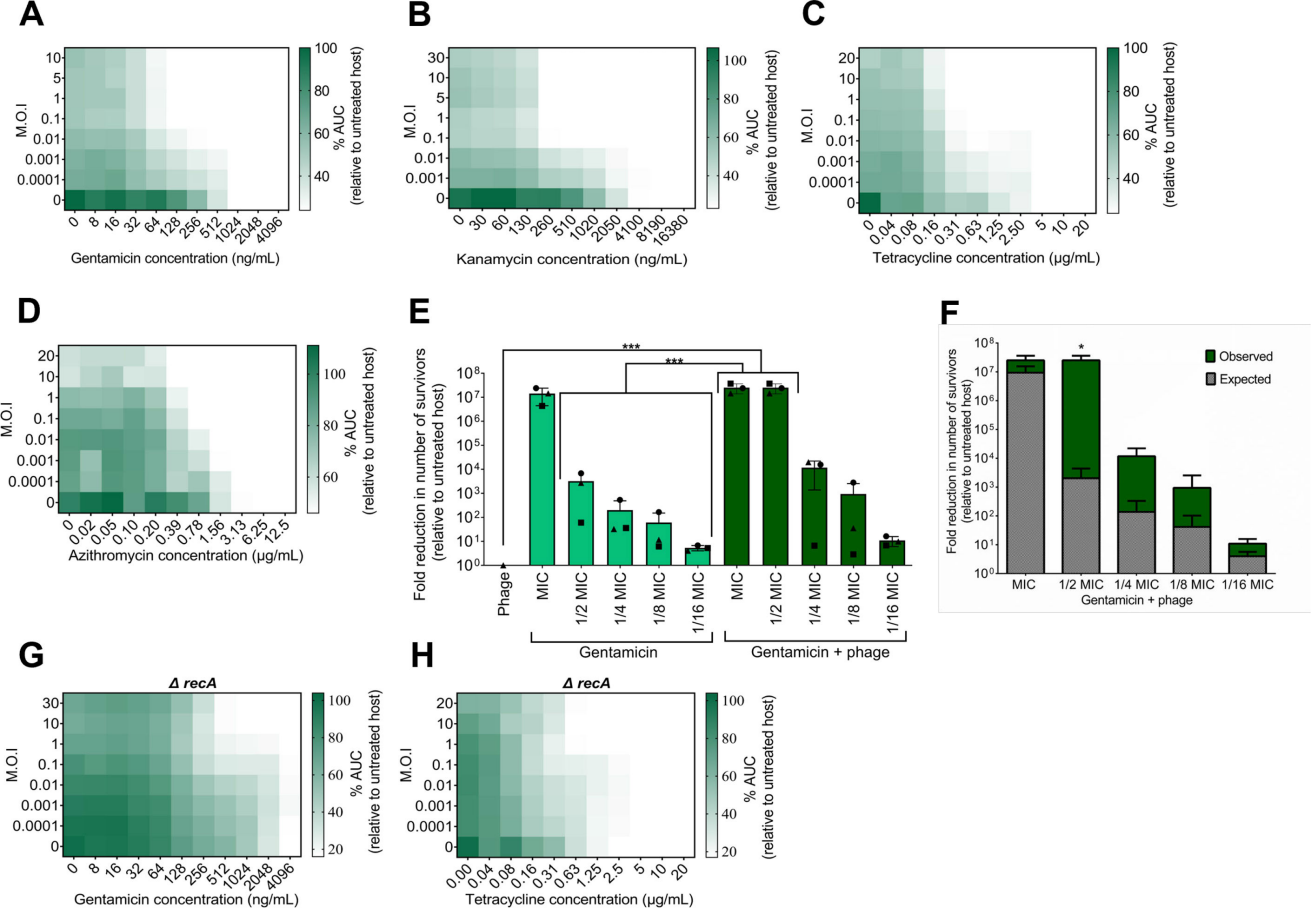
Protein synthesis inhibitors result in temperate phage-antibiotic synergy. Checkerboard assay of HK97 and (**A**) gentamicin, (**B**) kanamycin, (**C**) tetracycline, and (**D**) azithromycin. AUC relative to untreated bacterial control, averaged among three biological replicates, plotted as a heatmap. (**E**) Bars show the average number of survivors relative to untreated cultures in three biological replicates, each of three technical replicates. Each biological replicate is represented by its own shape: circle, square, or triangle. Limit of detection (10 CFU/mL) is represented at all points of MIC and 1/2 MIC tPAS data, as no counts were obtained, except “square” at 1/2 MIC. Error bars depict the SD, while ***P from 0.001 to 0.0001 from a one-way ANOVA and Tukey’s *post hoc* test. (**F**) Bars show the observed effect (green) versus the expected (gray) effect determined by multiplying the effect of the phage and antibiotic alone. Average from the three biological replicates for each observed tPAS data from Fig. 4E was compared to the calculated expected effect at the corresponding antibiotic concentration using a paired *t* test, *P ≤ 0.05. Checkerboard assay of HK97 and (**G**) gentamicin and (**H**) tetracycline in a *recA* mutant. Area under the curve relative to untreated bacterial control, averaged among three biological replicates, plotted as a heatmap.

We carried out checkerboards with two aminoglycosides, a tetracycline, and a macrolide. Unexpectedly, the use of HK97 with any of gentamicin, kanamycin, tetracycline, or azithromycin yielded a clear synergistic effect that resulted in efficient inhibition of bacterial growth (Fig. 4A through D). HK97 at MOI ≥ 0.1 resulted in an eightfold reduction in MIC of gentamicin, 16-fold with kanamycin or tetracycline, and eightfold reduction in MIC of azithromycin, although in the latter only at MOI ≥ 1. The use of protein synthesis inhibitors with temperate phage HK97 results in synergy through an unknown mechanism.

As this directly contradicts the findings of Kever et al. (77) with phage lambda, we repeated some of our protein synthesis inhibitor checkerboards in both lambda and its virulent mutant lambdavir. Unlike in their work, neither kanamycin nor gentamicin yielded antagonism with either phage (Fig. S5), although we also saw no synergy. Their work was done in aminoglycoside-resistant strains, with the antibiotic-modifying enzyme AphA1 responsible for the phosphorylation of the antibiotic. This could be separating a phage-synergizing effect (the antibiotic effect) from a lambda-specific inhibition by kanamycin. Alternatively, the discrepancy may be due to the different genotype of *E. coli* DSM613 (a B derivative) used in that study.

To quantify our synergy between protein synthesis inhibitors and HK97, we opted to focus on gentamicin, as it had the strongest synergistic effect at even very low phage concentrations. Survivors arising from the no-challenge, phage challenge, gentamicin challenge, and phage + gentamicin challenge were counted after overnight incubation in liquid media. This assay revealed bacterial eradication at MIC and 1/2 MIC gentamicin in combination with the phage. In a dose-dependent synergy, the killing effect decreased with decreasing antibiotic concentrations (Fig. 4E), with a more than four-log reduction in the number of survivors at 1/4 MIC compared to the untreated host. This synergy is up to 85-fold greater than the multiplicative effects of the phage and antibiotic alone (Fig. 4F).

As with the other antibiotics, we tested synergy in a *recA* mutant. As expected, neither gentamicin nor tetracycline synergy was dependent on RecA (Fig. 4G and H), although there did seem to be a requirement for a higher phage MOI to obtain comparable synergy. This is consistent with the literature (78) and with our findings that gentamicin did not detectably induce *recA* (Fig. S4).

### Mechanism of tPAS in protein synthesis inhibitors

**Fig 5:**
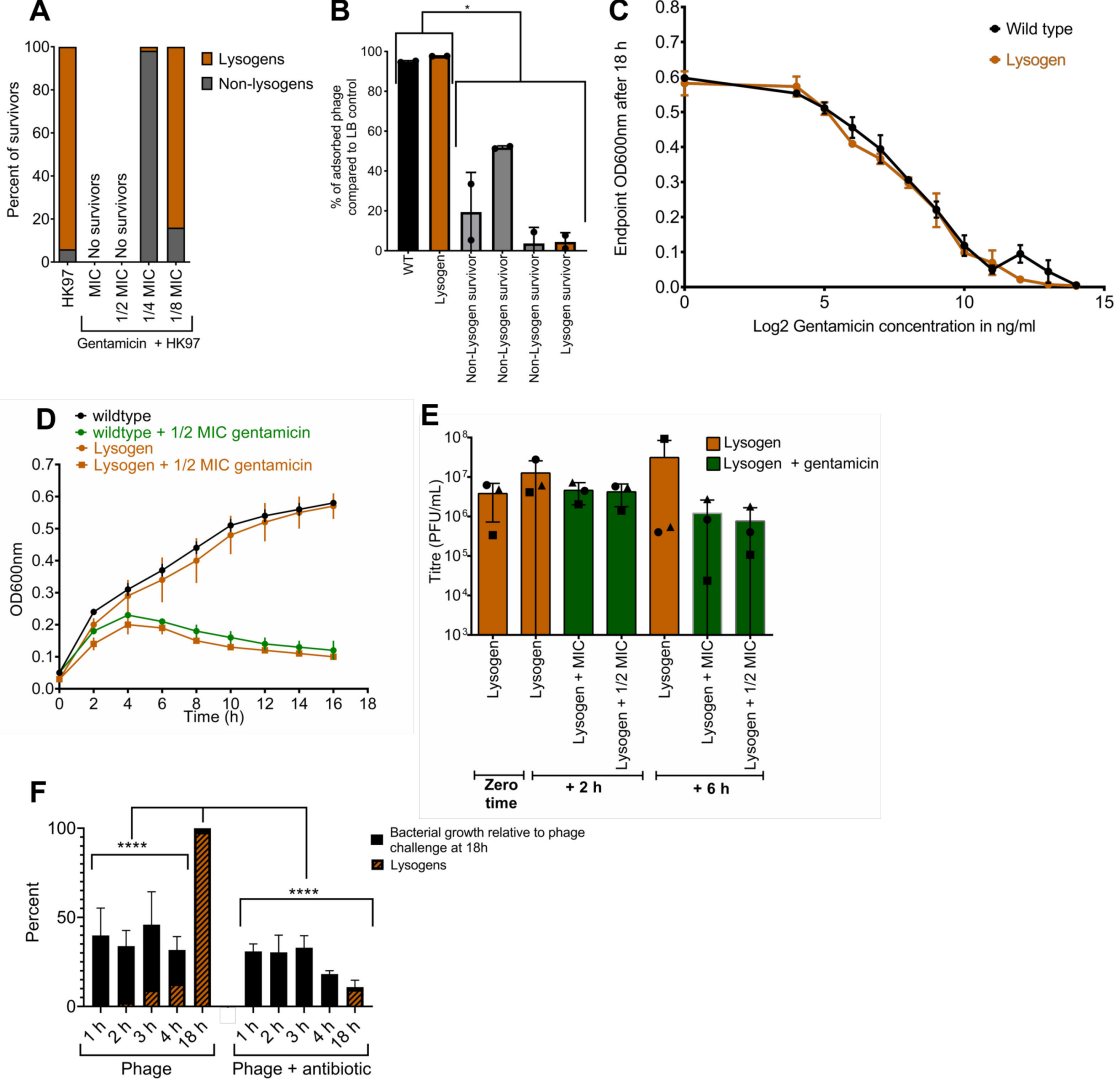
Mechanism of gentamicin HK97 synergy. (**A**) Percentage of lysogen and non-lysogen survivors after overnight HK97 and gentamicin challenges averaged. PCR was performed in duplicate for confirmation. (**B**) Phage adsorption of survivors. Bars showing the percentage of adsorbed phage in four survivors from 1/4 MIC gentamicin phage challenge; non-lysogens are in gray and orange is the single lysogen survivor. Error bars represent SD. Percent was compared using two-way ANOVA and Tukey’s multiple comparison tests, *P ≤ 0.05. (**C**) MIC in liquid culture for wild-type *E. coli* K-12, and lysogen control was tracked after challenging with serial dilutions of ciprofloxacin (means ± SD, *n* = 3). MIC is ~1.024 µg/mL for wild type and lysogen. (**D**) Growth curves in liquid culture of wild-type *E. coli* K-12 and lysogen control was tracked in the absence and presence of gentamicin at 1/2 MIC, averaged among three biological replicates ± SD. (**E**) Phage quantification at time 0 for lysogen control and after 2 and 6 h when challenged with serial dilutions of gentamicin, averaged among three biological replicates, each of three technical replicates. Error bars represent SD. Each biological replicate is represented by its own shape: circle, square, or triangle. (**F**) Percentage of lysogens and a representation of bacterial growth tracked over time for the HK97 or the HK97 and gentamicin challenge using qPCR at five time points (*n* = 3 performed in biological triplicates) for 1/2 MIC antibiotic. Significance of the results was studied using two-way ANOVA and Tukey’s multiple comparison *post hoc* test, ****P ≤ 0.0001.

Knowing this synergy is largely RecA independent, we had to determine whether the synergy obtained with protein synthesis inhibitors is tPAS, influencing the lysis-lysogeny decision. We first established whether the synergy was acting to reduce the frequency of lysogeny in survivors. Through PCR of the phage-host junction to confirm the integration of HK97, we screened purified survivors arising from the challenge at 1/4 MIC, where we started seeing our first survivors, as well as at 1/8 MIC. The antibiotic at 1/4 MIC reduced the percentage of lysogeny from 92% (*n* = 55 survivors) in the phage-alone challenge to 2% (*n* = 55) and reducing the antibiotic to 1/8 MIC restored lysogeny rates to 84% (*n* = 55) (Fig. 5A). These results show efficiency in lysogeny reduction that exceeds that in our previous work with ciprofloxacin (49).

Interestingly, the sole lysogen obtained at 1/4 MIC was also resistant to lambda-vir infection through altered adsorption (Fig. 5B). While surface receptor mutation is the most common resistance mechanism *in vitro* (79), and we expect it to be present in all tested non-lysogens (Fig. 5B). This is unusual in a lysogen because it should be protected from subsequent infections by preventing DNA entry through superinfection exclusion (80) or preventing gene expression via superinfection immunity conferred by the HK97 cI repressor. There should be no selective pressure on a lysogen to block the adsorption of the phage. We hypothesized that gentamicin might be reducing the effectiveness of superinfection immunity, but superinfections of lysogens in the presence of sub-inhibitory gentamicin yielded no plaques (not shown).

Next, we sought to establish whether gentamicin was inducing lysogens through some unknown pathway by comparing the antibiotic sensitivity of the lysogen to the parent bacterium. MICs were found to be indistinguishable between the lysogen and the parent bacterium (Fig. 5C), and no change in growth was observed upon exposure of the lysogen and non-lysogen to 1/2 MIC gentamicin (Fig. 5D). This was accompanied by no change in HK97 phage titer at 2 or 6 h post-exposure at both MIC and 1/2 MIC (Fig. 5E). Neither induction nor increases in burst size appear to be occurring.

Gentamicin clearly decreases the rate of lysogeny (Fig. 5A) but has no effect on the number of phages produced by a lysogen (Fig. 5E). Moreover, the lysogen has no increased sensitivity to gentamicin (Fig. 5C and D), a characteristic property of phage-inducing antibiotics. If gentamicin reduces the frequency of lysogens but, unlike ciprofloxacin, not by selecting against them, it must be instead biasing the initial lysis-lysogeny decision against lysogeny. To investigate this hypothesis, *E. coli* was challenged with HK97 with or without gentamicin at 1/2 MIC, incubated overnight, and sampled over time. Samples were treated with DNase to remove any extracellular DNA, whether phage or bacterial, from cells already lysed. We then extracted genomic DNA and followed the frequency of lysogeny over time with qPCR primers for the HK97-host junction. As early as the 2 h mark, the percentage of lysogens was significantly lower in the presence of the antibiotic, and this trend persisted out to the endpoint at 18 h (Fig. 5F). This is in direct contrast to ciprofloxacin, whose detectable effect on the ratio of lysogens to non-lysogens was only seen after 6 h (49), and supports our claim that gentamicin is biasing the initial lysis-lysogeny decision in an SOS-independent manner.

At the 18 h mark, using qPCR, the “survival rate” for the phage + antibiotic challenge at 1/2 MIC gentamicin is approximately 10% (Fig. 4F), contrasting with no survivors detected in the colony assay at the same concentration (Fig. 4A). This suggests that though DNase-protected bacterial genomes are detected at 18 h in the phage + antibiotic challenge (Fig. 4F), these genomes are associated with non-viable cells. This increased sensitivity of the qPCR assay relative to screening surviving colonies further bolsters our case, as even this extremely sensitive assay detected almost no lysogeny at early time points (0.03%, 0.4%, 1.8%, and 1.5%, over the first 4 h in order). Gentamicin suppresses lysogeny and skews the initial lysis-lysogeny decision. To extend our findings to other protein synthesis inhibitors, we performed the same qPCR-based assay using 1/2 MIC tetracycline and found the same pronounced initial inhibition of lysogeny (Fig. S6).

This is not the first report of factors that can force a lytic cycle outside the SOS response; overexpression of capsular polysaccharide synthesis genes *rcsA* and *dsrA* cause lambda and lambdoid prophage induction in a *recA* mutant (81), and induction can also be controlled by autoinducers (82), internal ionic environment (83), EDTA exposure (84), and micropollutants (85). Moreover, oral administration of commonly prescribed drugs and dietary products can induce phages, although the SOS response was not ruled out (86–88). However, all these studies revealed induction rather than the prevention of lysogeny. Interestingly, we have uncovered an entirely new SOS-independent way of manipulating phage behavior and biasing the initial lysis-lysogeny decision. Fortuitously, this results in potent tPAS. As this synergy appears to exist across aminoglycosides tested, as well as in tetracycline and azithromycin—other protein synthesis inhibitors—we suspect that it arises from a delay in the accumulation of lysogeny-favoring proteins—potentially CII (89), therefore greatly decreasing the likelihood of lysogeny.

### Conclusion

**Fig 6:**
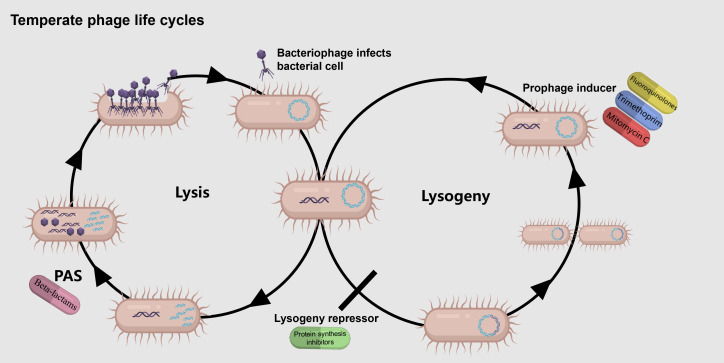
Antibiotics synergizing with temperate phages. Antibiotics that activate the SOS response act as prophage inducers and synergize with temperate phages (top right). Protein synthesis inhibitors also synergize with temperate phages but do so by blocking entry to lysogeny (bottom, center). While some β-lactams do show synergy with temperate phages, mechanistically this appears to be independent of the lysis-lysogeny decision (**L**) Created with BioRender.com.

This is the first demonstration of the broad applicability of temperate phage synergy with not only SOS-inducing but also non-SOS-inducing antibiotics (summarized in Fig. 6). We demonstrate that temperate phage HK97 can lower the effective MIC of antibiotics belonging to seven different drug classes. This could enable the use of temperate phages in therapy as adjuvants to antibiotics, serving as a “safety net” reaching maximum synergy if antibiotic concentrations fall—either due to dosing issues or the emergence of resistance—below MIC. This could also be done in combination with non-antibiotic inducers (e.g., reference 86). Excitingly, we uncovered the first way to block entry to lysogeny: protein synthesis inhibitors (Fig. 6, bottom-center). These do not act as phage inducers, and by enabling us to separate the entry into lysogeny from its exit, they provide us with a powerful tool to study the lysis-lysogeny decision—arguably the single most important decision point in microbiology—in-depth.

## MATERIALS AND METHODS

### Experimental model and subject details

The temperate phage models used for this study are lambdoid phage HK97 and lambda. Lambda(vir) was used as a lytic phage model. *E. coli* K-12 (Ymel mel-1 supF58) and the *E. coli* BW25113 *recA* mutant, obtained from the Dharmacon KEIO collection through Horizon Discovery (Cambridge, UK), are the two hosts. *recA* deletion was confirmed by PCR, using two sets of primers that bind inside and outside *recA* region (Fig. S7A), as well as profiling the antibiotic sensitivity of the strain (Fig. S4). Furthermore, we tested the ability of the strain to be infected by the phage, lysogenized as expected, and evaluated the rates of spontaneous induction expected of a *recA* mutant (Fig. S7B). Curiously, this highlighted considerable variability in underlying phenotypes (not shown). We selected lysogens with the lower rates of spontaneous induction reported in the literature and also confirmed the Δ*recA* lysogen’s lack of increased insensitivity to ciprofloxacin relative to the parental mutant (Fig. S7C). The *E. coli* K-12 host and phages were obtained from the Félix d’Hérelle Reference Center for Bacterial Viruses under the identifier HER 1382 and HER 382, respectively, with λ-vir (HER37) propagated on the same host. Bacterial culture was grown as previously described (48). Briefly, growth was in 10 mL lysogeny broth (LB) at 37°C with shaking at 130 rpm (Ecotron, Infors HT, Quebec, Canada). For same-day use, overnight cultures were diluted 1:100 in LB broth and grown to OD_600_ of 0.2, measured using the Thermo Fisher Scientific Spectronic 20D+ (Waltham, MA, USA).

### Method details

#### Phage propagation and titration

Phage lysates were obtained by primary amplification by inoculating frozen bacterial and phage stocks in 10 mL LB broth growing for a maximum of 18 h or secondary amplification by inoculating 50 µL of previously prepared phage lysate into 10 mL of grown culture followed by incubation at 37°C for up to 4 h. Cultures were then passed through a 0.45 µm Basix Syringe Filters, PES, Sterile from Thermo Fisher Scientific to obtain a phage lysate. Phage titration was carried out using the double agar overlay technique (90). A volume of 300 µL of overnight grown bacterial culture and 100 µL of 10-fold serial dilutions of the lysate prepared in LB were mixed into molten soft agar 0.75% (wt/vol) and distributed onto solid 1% (wt/vol) agar. Plaques were counted as zones of clearing in bacterial lawns after overnight incubation. Multiplicity of infection was determined using the following formula: phage titer (PFU/mL) × phage volume/colony-forming unit (CFU/mL) × bacterial volume (mL).

#### MIC determination

MIC of antibiotics was determined using a slightly modified broth dilution method (91). Briefly, 100 µL of freshly grown culture, a volume of antibiotic stock solution, and nuclease-free water were combined in a microtiter plate to obtain a final volume of 250 µL. The microtiter plate was incubated for 18 h at 37°C overnight with double orbital shaking at a frequency of 205 cpm (5 mm) using an Epoch 2 microplate spectrophotometer (BioTek Instruments, Inc., VT, USA). MIC determination was performed in triplicate, and endpoint OD_600_ was measured after 18 h. The MIC was the lowest concentration of antibiotic in which the final OD was equal to the initial read at time zero.

#### Checkerboard assay

One hundred microliters of cultures grown until an OD_600_ of 0.2 was transferred into wells in a 96-well plate containing 100 µL of previously diluted phage lysate in LB to achieve target MOIs on the vertical axis. In all our checkerboards, we started horizontally with the highest antibiotic concentration of at least MIC and then we performed a twofold serial dilution with a final volume of 250 µL. Synergy testing was performed in triplicate, in which the optical density was monitored every 15 min for 18 h using an Epoch 2 microplate spectrophotometer, followed by percent growth measurements calculated as follows: (OD_treatment_ − OD_growth control_/OD_growth control_) × 100. The results were graphically represented in a heatmap. Our threshold value for heatmaps was calculated for each antibiotic based on antibiotic-alone condition growth curves (see Fig. S1). Cutoff value was selected based on percent growth value relative to untreated host at MIC for endpoint checkerboards and percent AUC relative to untreated host value for timepoint measurement heatmaps.

#### Fluorescence assay

Two *E. coli* strains with engineered promoter-reporter gene construct that expresses GFP upon *recA or pitB* expression were obtained from the Brown Lab at McMaster University, which were originally obtained from reference 68. One hundred microliters of freshly grown cultures untreated or treated with MIC, 1/2 MIC, and 1/4 MIC concentrations of antibiotics (ciprofloxacin, levofloxacin, mitomycin C, ceftazidime, trimethoprim, and gentamicin) was added to a 96-well microplate for fluorescence-based assays. Twenty-five microliters of the volume of kanamycin 500 µg/mL stock solution was added for strain selection and nuclease-free water was combined in a microtiter plate to obtain a final volume of 250 µL. The microtiter plate was incubated for 18 h at 37°C overnight with double orbital shaking at a frequency of 205 cpm (5 mm) using Agilent BioTek Synergy Neo2 multimode microplate reader (BioTek synergy Neo2 Instruments, Inc., VT, USA). The resulting fluorescence was initially normalized to OD growth. We then calculated fold change relative to fluorescence in the untreated host. Normalized fold change in *recA* fluorescence was then plotted relative to the average normalized fold change in *pitB* in three technical replicates.

#### Broth growth curve

Growth curves in liquid culture for challenged and non-challenged wild-type *E. coli* K-12 and *E. coli* K-12 HK97 lysogen were recorded as follows. Freshly grown cultures were treated with 1/2 MIC concentration of gentamicin (512 ng/mL). Cultures were then incubated overnight with double orbital shaking, and readings were taken every 15 min with the Epoch 2 microplate spectrophotometer.

#### Overnight quantification assay

In a 1.5 mL microcentrifuge tube, 100 µL of freshly grown cultures, 100 µL of phage lysate for a final MOI of at least 10, and twofold serial dilutions of antibiotics were added to a final volume of 350 µL and mixed by pipetting. Cultures were then incubated overnight with shaking at 130 rpm (Ecotron, Infors HT, Quebec, Canada). Subsequently, a 10-fold serial dilution of each trial was prepared, inoculated in 5 mL of LB soft agar (0.75%), and then incubated overnight. Survivors from each challenge were counted, and the actual number of survivors in 1 mL broth was calculated. Subsequently, fold reduction compared to the untreated host was calculated as follows: actual count of untreated host/actual count of each challenge, and then expected synergy was calculated as follows: fold reduction of phage challenge × fold reduction of each antibiotic challenge.

#### Adsorption assay

A volume of 1 mL of either freshly grown purified survivor cultures, lysogen control, or LB broth control was incubated with shaking for 30 min with 100 µL of diluted lambda-vir phage lysate of titer 10^4^ pfu/mL. Subsequently, 100 µL of each tube after filtration by centrifugation was mixed with 300 µL of host overnight culture, inoculated in 5 mL of molten LB soft agar, and then overlay plates were prepared. Plaques were counted from plates after an overnight incubation at 37°C. The percentage of adsorbed phages was then calculated as follows: (plaque count of blank − plaque count of each sample) × 100/phage plaque count of blank.

#### Phage titer after lysogen challenge with antibiotic

The number of phage particles arising from lysogens in the absence of antibiotics and in the presence of antibiotics at two concentrations, MIC and 1/2 MIC, was determined using a phage plaque assay. Freshly grown lysogen cultures with and without antibiotics were filtered using 0.45 µm Basix Syringe Filters, PES, Sterile from Thermo Fisher Scientific to obtain phage lysates. This was done at time 0, 2, and 6 h. A serial dilution of 10-fold was carried out in LB in a final volume of 1 mL. Lysates were titered using the standard double agar overlay technique. After overnight incubation, plaques were counted to calculate the number of phage particles in pfu/mL.

#### Βeta-lactams’ induction assay

Phage induction assay was carried out using wild-type *E. coli* K-12 and *E. coli* K-12 HK97 lysogen. One hundred microliters of overnight culture was inoculated into 10 mL LB broth and grown to an OD of 0.2. In a 96-well plate, 100 µL of culture was challenged with twofold serial dilution of antibiotic in a final volume of 250 µL. The plate was incubated with a porous adhesive seal in a 130 rpm incubator at 37°C for 18 h. Endpoint growth (OD_600_) was measured using the Epoch 2 microplate spectrophotometer (BioTek 432 Instruments, Inc., VT, USA). The plate was filtered using the Millipore MultiScreenHTS vacuum manifold (cat. MSVMHTS00, Darmstadt, Germany) with a Millipore Sigma MultiScreenHTS High Volume 96-well 0.45 µm filter plate (cat. VHVN4525, Darmstadt, Germany). To quantify phages, lysates of wild-type and HK97 lysogen with 1/2 MIC and zero antibiotics were diluted serially 10-fold in LB broth, and 3 µL was spotted on wild-type sensitive host using the double agar overlay method (300 µL of overnight culture added into molten 3 mL of 0.75% LB agar poured onto 1% LB agar). Phage titer was quantified after incubating plates overnight at 37°C.

#### Lysogeny detection

The integration of HK97 into the host chromosome was confirmed via polymerase chain reaction (PCR) two times for confirmation. Individual surviving colonies arising from the PAS challenge with 1/4 and 1/8 MIC, where we started seeing our first survivors, were purified by streaking. This was followed by colony PCR in which primers were designed to amplify the phage-host junction. Each 25 µL PCR reaction contained 1 mL of each primer, 2.5 mL 10× DNA polymerase buffer, 0.5 mL dNTPs, 0.25 mL Taq DNA polymerase, and 1 µL of purified survivor grown overnight, and the remaining volume was completed with nuclease-free water. All PCR reagents were obtained from FroggaBio (NY, USA). Primer sequences are available in key resources table (Table 1) (48).

**TABLE 1:** Primers used in this study

Oligonucleotides	Source	Notes
Primers for lysogen detection attBF: TGAATCCGTTGAAGCCTGCT	This paper	N/A
HK97_lys_R: GCGTGTAATTGCGGAGACTT	This paper	N/A
Primers for non-lysogen detection in qPCR attBF-veR: GCCTCGATTACTGCGATGTTTAG	This paper	Used with attBF to detect non-lysogens in qPCR
Primers for lysogen detection in qPCR HK97_lys_R2: CGTGATGACAGAGGCAGGG	This paper	Used with attBF to detect lysogens in qPCR
Primers for *E. coli* cysG detection in qPCR CysG_F2: AGGGGTTTTTACGTGGATCATTTG	This paper	N/A
CysG_R2: GGTGAACTGTGGAATAAACGCT	This paper	N/A
Primers that bind inside recA region: recA_F: GTCAACCAGTTCGCCGTAGA	This paper	Used to confirm *recA* deletion
Primers that bind inside recA region: recA_R: GGGCCGTATCGTCGAAATCT	This paper	
Primers that bind outside recA region recA_fwd: CGGTATTACCCGGCATGACA	This paper	
Primers that bind outside recA region: recA_rev: GCAGATGCGACCCTTGTGTA	This paper	

#### Βeta-lactam challenge lysogeny detection

Twenty colonies that survived overnight challenges with HK97 and HK97 + 1/4 and 1/16 MIC antibiotic were picked and streak purified three times on LB 1% agar plates. Purified colonies were inoculated into 250 µL LB broth in a 96-well plate and incubated overnight at 37°C. Wild-type *E. coli* K-12, an *E. coli* K-12 HK97 lysogen, and LB broth were added as controls. Cultures were stamped with a disposable 96-pin replicator (V&P Scientific, Inc, cat. VP 246, San Diego, CA, USA) onto a rectangular Nunc OmniTray LB 1% agar plate (cat. 242811, Thermo Scientific, Rochester, NY, USA) with a soft agar overlay of *E. coli* K-12 (10 mL 0.75% agar + 1 mL of overnight culture). Cultures were also stamped onto LB 1% agar plate, with no overlay, as a growth control. After overnight incubation at 37°C, survivors that resulted in a zone of clearing around the stamped spot on wild-type host were categorized as lysogens with baseline spontaneous induction. The frequency of lysogeny was calculated as the percentage of the total number of survivors that were characterized as lysogens.

#### Quantitative PCR

qPCR was carried out as previously described (48). Briefly, freshly grown culture was challenged with phage at an MOI of at least 10 in the absence and presence of 1/2 MIC gentamicin in three biological replicates, each with five replicates. Challenges were incubated with shaking at 37°C and 130 rpm (Ecotron, Infors HT, Quebec, Canada). One replicate of each of the two challenges was removed after 1, 2, 3, 4, and 18 h of exposure. To remove free-floating DNA from lysed cells, challenges were treated with DNase, followed by the addition of EDTA and heat inactivation at 75°C for 10 min. Genomic extraction was carried out using the Monarch Genomic DNA Purification Kit (New England Biolabs, MA, USA). The *E. coli* housekeeping gene *cysG* was used as a control for the quality of the DNA extraction in that sample. Each sample was amplified using primers designed to detect *cysG*, Hk97 lysogen integration site, and non-lysogens. PowerUp SYBR Green Master Mix (Applied Biosystems, MA, USA), BioRad CFX96 Touch Real Time Detection System, and CFX Manager 3.1.1517.0823 (CA, USA) were used to carry out qPCR. qPCR cycling mode was as follows: initial denaturation at 94°C for 2 min. This was then followed by 40 cycles as follows: denaturation at 95°C for 15 s and annealing/extension at 60°C for 1 min. The melt curve was generated by heating from 65°C to 95°C in 0.5°C increments per second. Primer sequences are available in key resources table (Table 1) (48).

### Quantification and statistical analysis

All the statistical details of experiments can be found in the figure legends, figures, and results. Quantitative values were expressed by mean ± SD. They were compared by *t* test, one-way ANOVA, two-way ANOVA, and Tukey’s *post hoc* when appropriate, with *P* value ≤ 0.05 is considered significant. All statistical analyses were done using GraphPad Prism 9.2.0 (GraphPad Software, Inc., CA, USA).

## SUPPLEMENTAL MATERIAL

Fig. S1 mbio.00504-24-s0001.tif Endpoint readings for wild type antibiotic challenges.

Fig. S2 mbio.00504-24-s0002.tif Representation of endpoint vs area-under-curve checkerboards.

Fig. S3 mbio.00504-24-s0003.tif Endpoint readings for *recA* mutant antibiotic challenges.

Fig. S4 mbio.00504-24-s0004.tif *recA* and *sulA* reporter fluorescence.

Fig. S5 mbio.00504-24-s0005.tif Checkerboard assays for lambda and lambdavir.

Fig. S6 mbio.00504-24-s0006.tif Bacterial growth and percent of lysogens.

Fig. S7 mbio.00504-24-s0007.tif Confirmation of Δ*recA* lysogen.

Legends mbio.00504-24-s0008.docx Legends for Fig. S1-S7.

Supplemental tables mbio.00504-24-s0009.xlsx Raw data tables.

ASM does not own the copyrights to Supplemental Material that may be linked to, or accessed through, an article. The authors have granted ASM a non-exclusive, world-wide license to publish the Supplemental Material files. Please contact the corresponding author directly for reuse.
