# Supplementary figures and images for: Temperate phage-antibiotic synergy across antibiotic classes reveals new mechanism for preventing lysogeny
Source: mBio. 2024 May 17;15(6):e00504-24. doi: 10.1128/mbio.00504-24 (PMC11237771; doi:10.1128/mbio.00504-24)

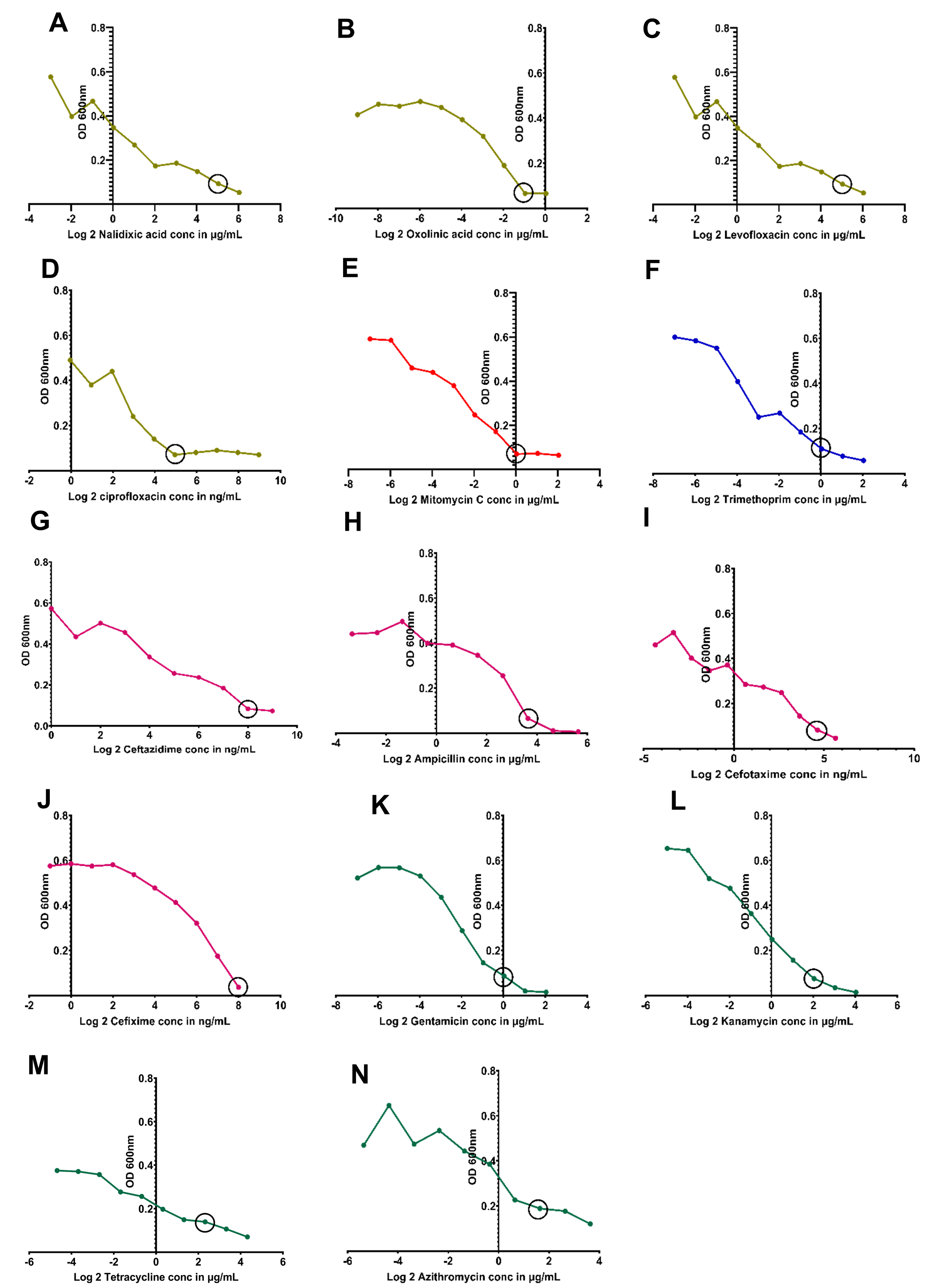

Supplement: Fig. S1 — Endpoint readings for wild type antibiotic challenges. [file mbio.00504-24-s0001.tif]

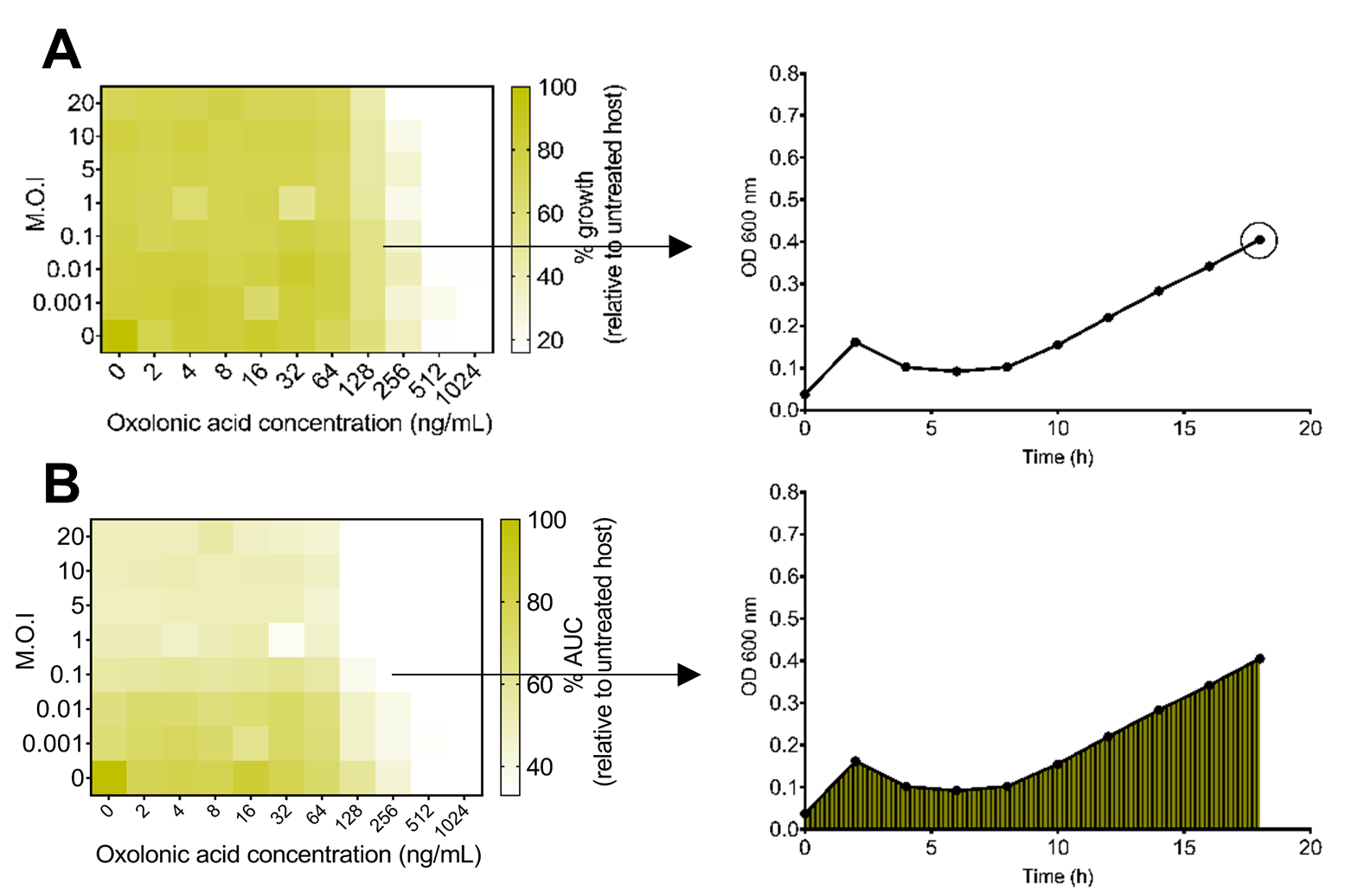

Supplement: Fig. S2 — Representation of endpoint vs area-under-curve checkerboards. [file mbio.00504-24-s0002.tif]

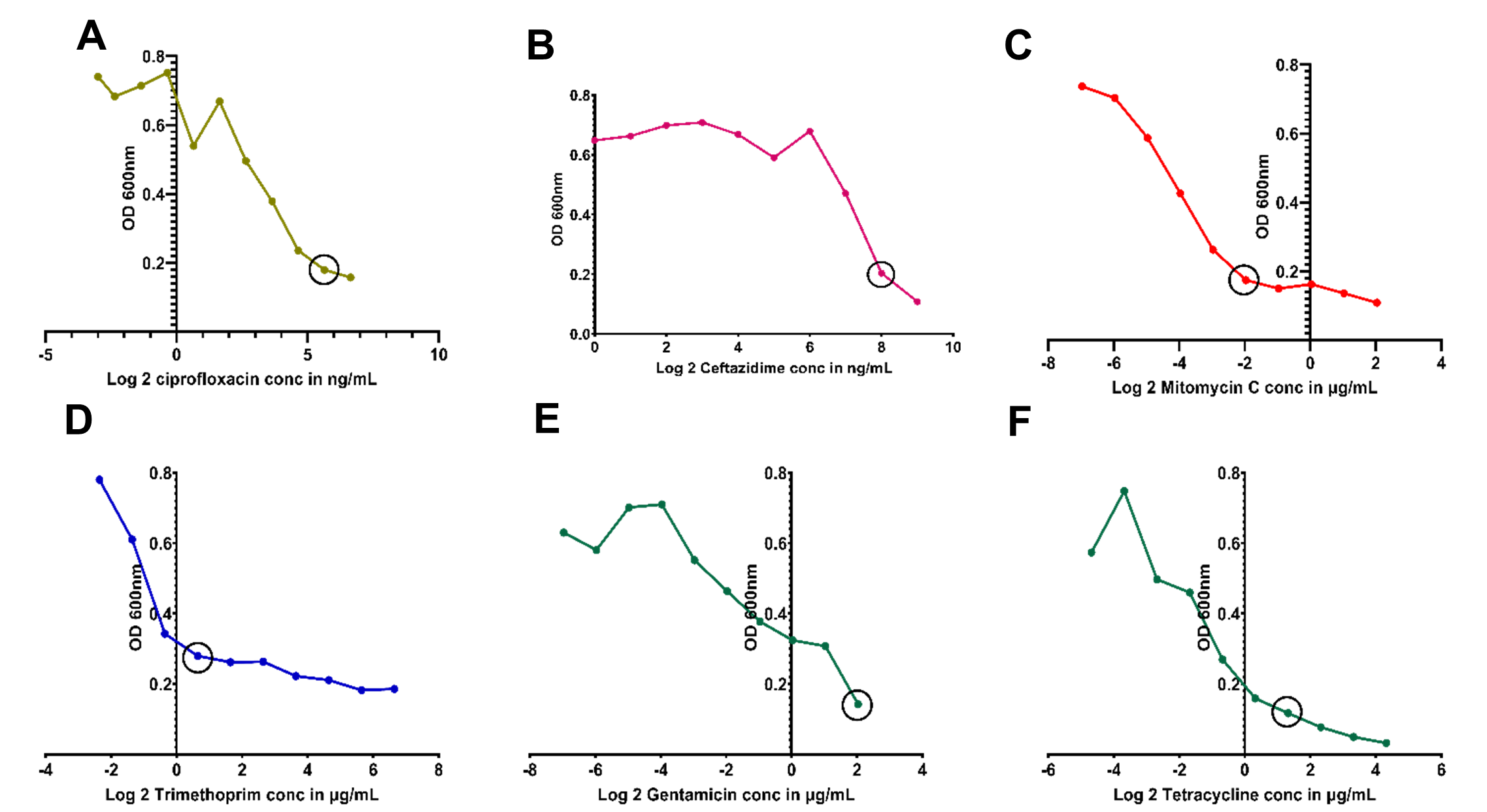

Supplement: Fig. S3 — Endpoint readings for recA mutant antibiotic challenges. [file mbio.00504-24-s0003.tif]

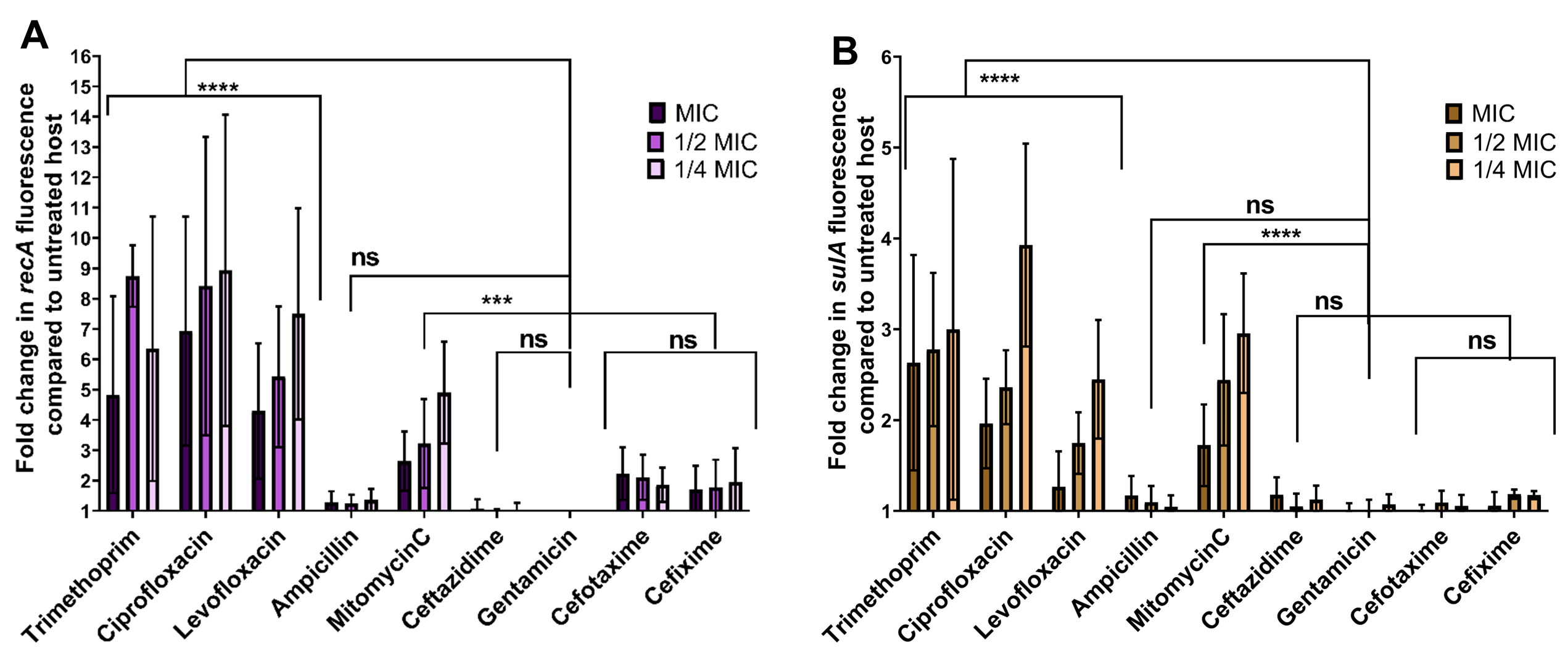

Supplement: Fig. S4 — recA and sulA reporter fluorescence. [file mbio.00504-24-s0004.tif]

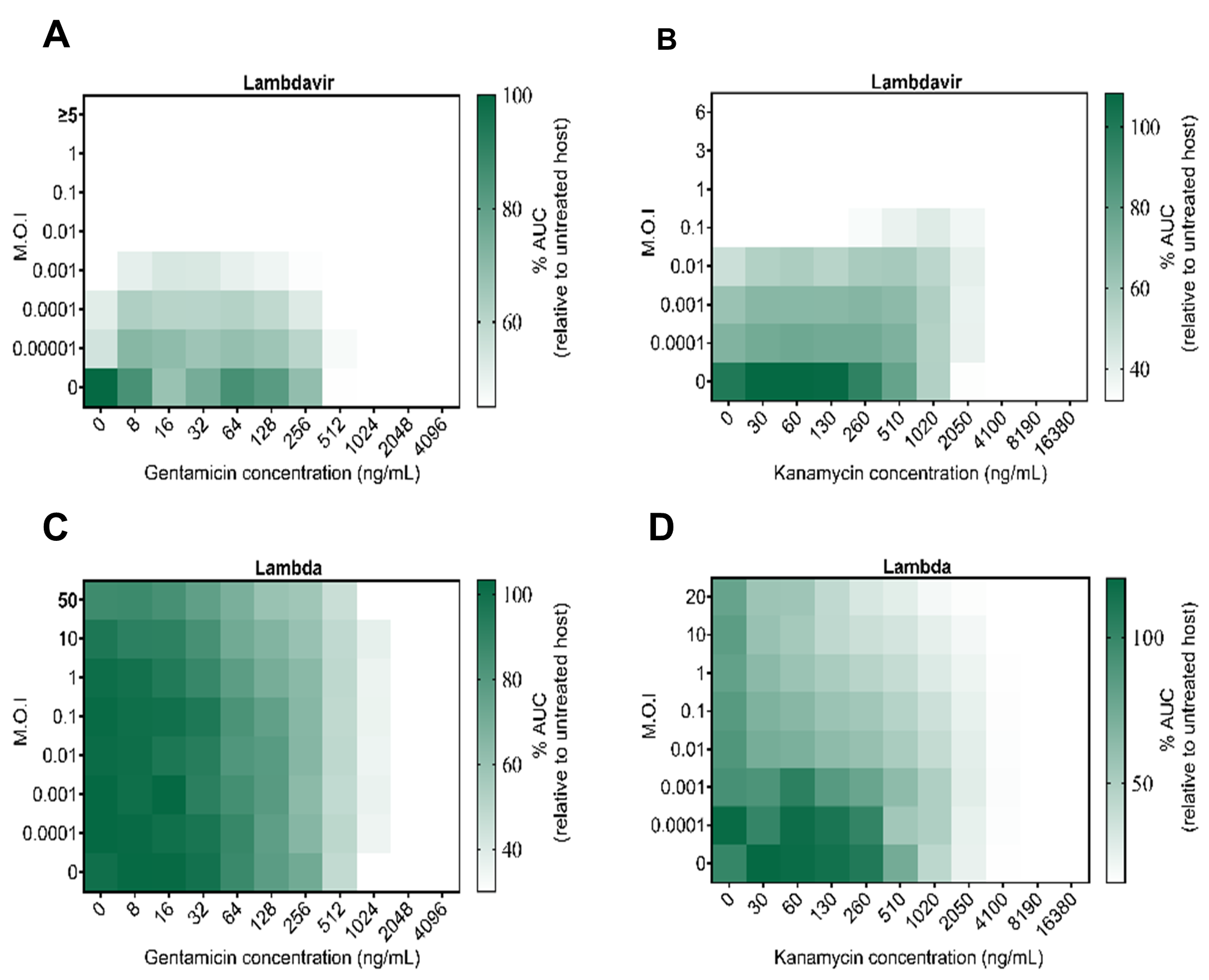

Supplement: Fig. S5 — Checkerboard assays for lambda and lambdavir. [file mbio.00504-24-s0005.tif]

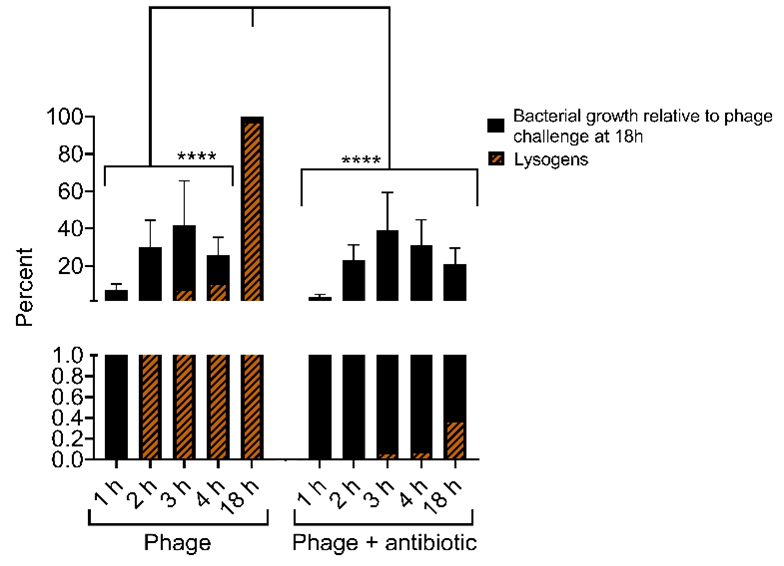

Supplement: Fig. S6 — Bacterial growth and percent of lysogens. [file mbio.00504-24-s0006.tif]

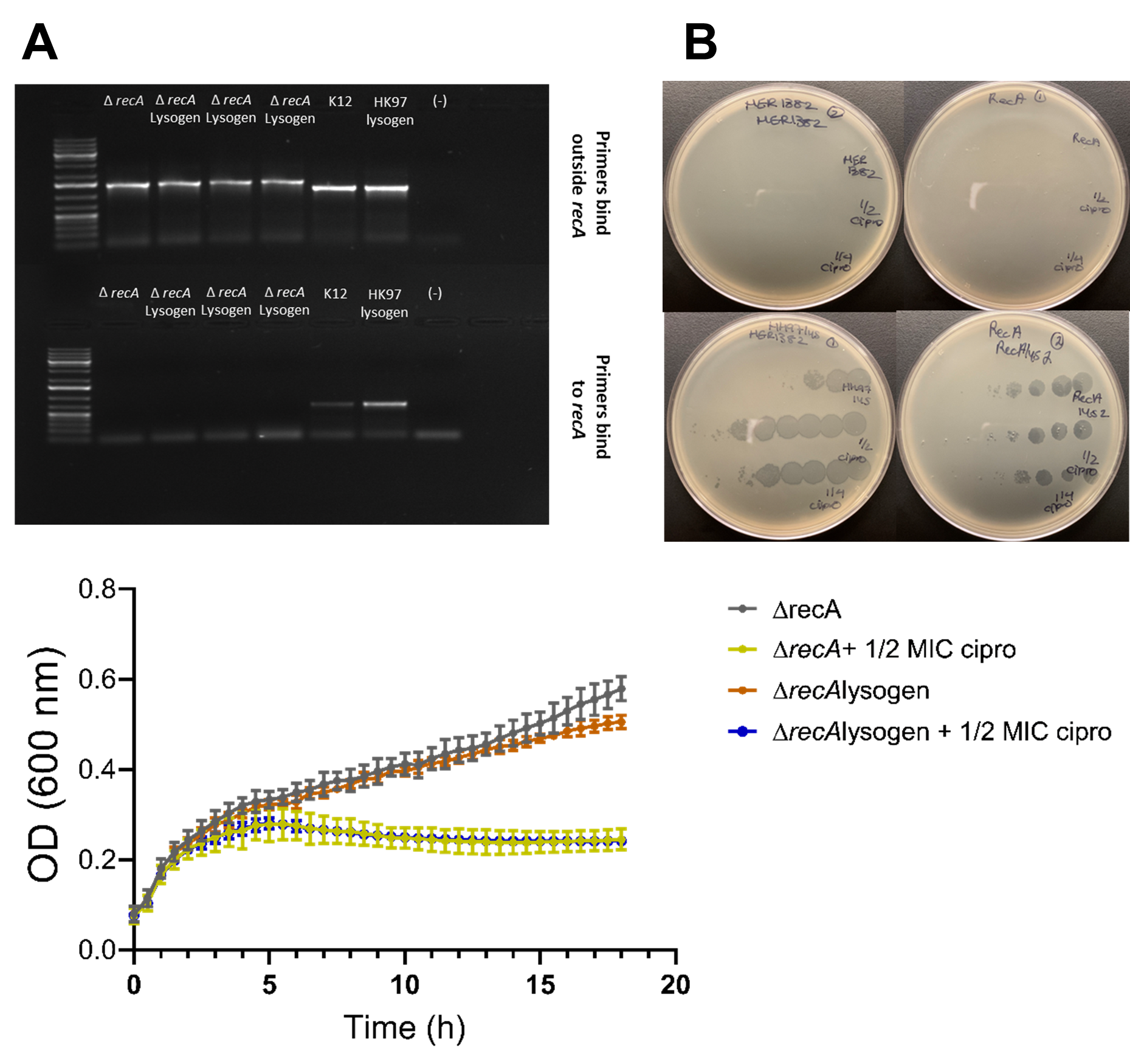

Supplement: Fig. S7 — Confirmation of ΔrecA lysogen. [file mbio.00504-24-s0007.tif]
