# Supplementary material for: Temperate phage-antibiotic synergy across antibiotic classes reveals new mechanism for preventing lysogeny
Source: mBio. 2024 May 17;15(6):e00504-24. doi: 10.1128/mbio.00504-24 (PMC11237771; doi:10.1128/mbio.00504-24)
Supplement: Legends — for Fig. S1-S7. [file mbio.00504-24-s0008.docx]

Fig S1.Endpoint readings after 18 hours of wild-type *E. coli* K-12 tracked in the absence and presence of serial concentrations of **(A)** Nalidixic acid, **(B)** Oxolinic acid, **(C)** Levofloxacin, **(D)** Ciprofloxacin, **(E)** Mitomycin C, **(F)** Trimethoprim, **(G)** Cefotaxidime, **(H)** Ampicillin, **(I)** Cefotaxime, **(J)** Cefixime, **(K)** Gentamicin, **(L)** Kanamycin, **(M)** Tetracycline and **(N)** Azithromycin averaged among three biological replicates. MIC is labelled in black circle.

Fig S2. Illustration if the difference between **(A)** heat map based on end point reading and growth curve at MOI of 1 and oxolinic acid concentration of 256 ng/mL **(B)** heat map based on time point readings (AUC) and growth curve at the same point illustrating AUC. Both for oxolinic acid challenged with HK97.

Fig S3. Endpoint readings after 18 hours of *recA* mutant tracked in the absence and presence of serial concentrations of **(A)** Ciprofloxacine **(B)** Cefotaxidime, **(C)** Mitomycin C, **(D)** Trimethoprim, **(E)** Gentamicin and **(F)** Tetracycline averaged among three biological replicates. MIC is labelled in black circle

Fig S4. *recA* and *sulA* reporter fluorescence fold change **(A)** recA reporter fluorescence fold change. Bars show average fold change in recA reporter fluorescence relative to untreated host in 3 biological replicates, each with 3 technical triplicates. **(B)** sulA reporter fluorescence fold change. Bars show average fold change in sulA reporter fluorescence relative to untreated host in 3 biological replicates, each with 3 technical triplicates. Note that the y-axis starts at 1; in both graphs, over 1-fold change would indicate an increase in normalized fluorescence compared to untreated host. Fold change in three biological triplicates with error bars depicting the SD was compared using 2-way ANOVA and Tukey multiple comparison tests with ns indicates no significant difference.

Fig S5. Checkerboard assay of lambdavir and: **(A)** gentamicin and **(B)** kanamycin. Area under the curve relative to untreated bacterial control, averaged among 3 biological replicates, plotted as a heatmap. Checkerboard assay of lambda and: **(C)** gentamicin and **(D)** kanamycin. Area under the curve relative to untreated bacterial control, averaged among 3 biological replicates, plotted as a heatmap.

Fig S6. Percent of Lysogens and a representation of bacterial growth tracked over time for the HK97 or the HK97 and tetracycline challenge using qPCR at five time points (n = 3 performed in biological triplicates) for ½ MIC antibiotic. The results' significance was calculated using two-way ANOVA ****p ≤ 0.0001.

Fig S7. Confirmation of Δ*recA* Lysogen. (A) PCR confirmation of deletion of *recA* gene. Primers used in the top panel bind outside the *recA* gene and primers used in the bottom panel bind within the gene. (B) Serial dilutions of K12, HK97 lysogen, Δ*recA* or Δr*ecA* lysogens plated on K12 or Δ*recA*. (C) Growth curves in liquid culture of Δ*recA* or Δ*recA* lysogens tracked in the absence and presence of ciprofloxacin at ½ MIC, averaged among 3 biological replicates ± SD.

Note: Supplementary Tables in Raw Data.xls : This excel file contains all data used to generate the figures in this paper, with each tab of the file named after the figure panel in which the data were used.
